# Artificial oxygen carriers: a new future?

**DOI:** 10.1186/s13054-018-1949-5

**Published:** 2018-02-23

**Authors:** Donat R. Spahn

**Affiliations:** Institute of Anesthesiology, Anesthesiology - Intensive Care Medicine - OR-Management, University of Zurich and University Hospital Zurich, Raemistrasse 100, CH-8091 Zurich, Switzerland

**Keywords:** Artificial oxygen carriers, Anemia, Surgery, Ischemia, Contrast agent

Despite the fact that allogeneic red blood cell (RBC) transfusions can be life-saving in exsanguinating trauma patients, many adverse events impacting patient outcome have documented [1]. Therefore, artificial oxygen carriers were initially been developed as “blood substitutes” in the 1980s and 1990s. Artificial oxygen carriers can be grouped into hemoglobin-based oxygen carriers (HBOCs) and perfluorocarbon-based oxygen carriers (PFCs) [2].

The clinical use of artificial oxygen carriers has mainly been studied in trauma and major surgery [3]. HBOC studies in general did not show a benefit in the primary outcome parameter such as avoidance/reduction of RBC transfusions or 28-day mortality [3] and signs of vasoconstriction/hypertension due to nitric oxide scavenging and increased relative risk of myocardial infarction and death were shown in a meta-analysis [4]. Consequently, the Food and Drug Agency in 2008 put all HBOC trials on hold.

PFC studies in non-cardiac surgery were successful in reversing physiologic transfusion triggers and in reducing the need for allogeneic RBC transfusions [5]. In addition, there were no major safety issues. However, a PFC study in cardiac surgery was prematurely stopped due to an increased incidence of neurologic adverse events, and this program has never been re-started.

In recent years, focus on the potential clinical use of artificial oxygen carriers moved away from “blood substitutes” towards “oxygen therapeutics”. Due to the relatively short half-life of 12–24 h, this may indeed be reasonable. However, clinical studies showing clear benefits in this new area are still scarce. The area with most documented evidence are “compassionate use” programs. In such programs, patients were treated with HBOCs at a median hemoglobin concentration of 39 g/l [6]. Survival of patients with severe anemia for whom RBC transfusion was not an option was clearly and significantly higher if treated with an HBOC [7]. It is also conceivable that an HBOC may be capable of bridging a patient with severe anemia until RBC transfusions become available.

In animal models, artificial oxygen carriers have also proven to be efficacious in relieving organ ischemia such as fetal hypoxia in pre-eclampsia [8] and cerebral ischemia [9]. However, in a recent study myocardial perfusion with an oxygenated HBOC-enriched solution did not reduce the infarct volume nor was post-ischemic cardiac function improved [10]. In contrast, HBOC attenuated intense exercise-induced cardiac dysfunction [11].

Machine perfusion of liver grafts after prolonged cold ischemia with HBOC enriched perfusate appears to be efficacious in improving the condition of the liver graft prior to transplantation in multiple animal experiments [12]. And recently the first human liver transplantation after machine perfusion with HBOC was performed.^1^ The use of artificial oxygen carriers in pre-transplant perfusion is also conceivable in other organs such as lung and heart. The future will tell whether HBOCs or PFCs are more efficacious.

PFCs may also be used as contrast agents [13] and, in conjunction with magnetic resonance imaging, as infection tracers [14].

Finally yet importantly, artificial oxygen carriers look like a logical adjunct to Patient Blood Management. Patient Blood Management is already highly successful: a reduction in the use of allogeneic blood product transfusion of approximately 40%, a decrease in hospital mortality (−28%), infection rate (−21%), combined myocardial infarction and stroke (−31%), length of hospital stay (−15%), and annual costs ($7–29 million) has been described in a study on 605,000 patients in Western Australia [15]. Nevertheless, having an artificial oxygen carrier to bridge the period of low hemoglobin/hematocrit or in the context of augmented hemodilution [5] might broaden the spectrum of Patient Blood Management and may make it even more successful.

Artificial oxygen carriers thus may indeed have a new future in a large variety of clinical scenarios and diagnostic/therapeutic concepts.

## Footnotes

1 https://www.prnewswire.com/news-releases/hemoglobin-oxygen-therapeutics-llc-announces-the-worlds-first-human-liver-transplantation-after-ex-situ-normothermic-machine-perfusion-using-hemopure-300538456.html
